# Research on surrogate model of dam numerical simulation with multiple outputs based on adaptive sampling

**DOI:** 10.1038/s41598-023-38590-z

**Published:** 2023-07-24

**Authors:** Jiaming Liang, Zhanchao Li, Litan Pan, Ebrahim Yahya Khailah, Linsong Sun, Weigang Lu

**Affiliations:** 1 College of Water Resources Science and Engineering, Yangzhou University, Yangzhou, 225009 Jiangsu China; 2 Intelligent Water Conservancy Research Institute, Nanjing Jurise Engineering Technology Co., Ltd, Nanjing, 210032 Jiangsu China; 3 Huadian Electeric Power Research Institute Co., Ltd, Hangzhou, 310000 China; 4 Civil Engineering Department, College of Engineering, Thamar University, Dhamar, 504408 Yemen

**Keywords:** Civil engineering, Hydrology

## Abstract

Dam numerical simulation is an important method to research the dam structural behavior, but it often takes a lot of time for calculation when facing problems that require many simulations, such as structural parameter back analysis. The surrogate model is widely used as a technology to reduce computational cost. Although various methods have been widely investigated, there are still problems in designing the surrogate model's optimal Design of Experiments (DoE). In addition, most of the current DoE focuses on establishing a single-output problem. Designing a reasonable DoE for high-dimensional outputs is also a problem that needs to be solved. Based on the above issues, this research proposes a sequential surrogate model based on the radial basis function model (RBFM) with multi-outputs adaptive sampling. The benchmark function demonstrates the applicability of the proposed method to single-input & multi-outputs and multi-inputs & multi-outputs problems. Then, this method is applied to establishing a surrogate model for dam numerical simulation with multi-outputs. The result demonstrates that the proposed technique can be sampled adaptively and samples can be targeted based on the function form of the surrogate model, which significantly reduces the required sampling and calculation cost.

## Introduction

Almost for all countries in the world, dams are a vital part of the nation’s infrastructure^1^. In terms of flood control, power generation, irrigation, water supply, etc. The dam has yielded significant social and economic benefits. As a highly complex system, a large number of numerical simulations are required in the process of parameter back analysis, uncertainty analysis, sensitivity analysis, optimization design, and so on. Meanwhile, a large number of simulations bring extremely expensive computing cost. To overcome this issue, a surrogate model is used to replace the high-fidelity simulation model.

The surrogate model, also known as a meta-model^2^, is a simplified model with a small computation scale, but the calculation results are close to those of a model with high precision. The commonly used surrogate model includes the polynomial regression surface (PRS)^3–5^, Kriging model^6,7^, neural network model^8–11^, support vector regression (SVR) model^12–15^, radial basis function model (RBFM)^16–19^, and so on. RBFM is a function of the distance between data points, and has the characteristics of dimensionality independent and meshless. RBFM, as a precise interpolation model, not only performs well in interpolation accuracy but also has a relatively simple method with clear principles, requiring few specified parameters and easy operation. Therefore, it is extensively acknowledged in the field of surrogate model research^20–23^.

In recent years, many scholars have applied the surrogate model to dam research. Li et al.^24^ introduced a surrogate-assisted stochastic optimization inversion (SASOI) algorithm to identify the static and dynamic parameters based on Latin hypercube sampling (LHS). Shahzadi et al.^25^ constructed a surrogate model by combining polynomial chaos expansion and deep learning networks to assess the effect of constitutive soil parameters on the behavior of a rockfill dam using Sobol sampling. By combining the genetic algorithm (GA) with an updated Kriging surrogate model (UKSM), Wang et al.^26^ presented a new optimization procedure to reduce the computational cost for determining the optimal shape of a gravity dam based on LHS. Zhang et al.^27^ took the multi-layer perceptron algorithm as a surrogate model for determining deformation monitoring indexes based on LHS. Rad et al.^28^ constructed a new surrogate model by combining a hybrid SVR based on generalized normal distribution optimization (GNDO) with the Monte Carlo Simulation (MCS) to solve the RBDO problem of gravity dam design.

In the current research, LHS or Sobol sampling or other uniform sampling techniques are utilized more frequently to infill the design space. As it is widely acknowledged that in the absence of any priori knowledge on the problem under consideration, uniform sampling of the design space throughout the whole space is favourable. This kind of Design of Experiments (DoE) of sampling the sample space once is called one shot sampling method (OS). Methods such as central composite design^29^, uniform design^30^, LHS^31^, Generalized LHS^32^, and optimal Latin hypercube sampling (OLHS)^33^ are frequently employed. However, these methods often have high uncertainty in the high-nonlinear region, resulting in bad model performance^34–36^. Especially when dealing with a complex and high-dimensional dam structure, establishing the dam surrogate model contains numerous regions of high uncertainty. Therefore, DoE based on the aforementioned strategies frequently requires many samples in order to establish a sufficiently accurate surrogate model.

The adaptive sampling strategy is applied to DoE to obtain a sufficiently accurate surrogate model with a minimum sample capacity,. Under this strategy, the present surrogate model is used to assist the selection of the new samples, to improve the model's local optimization or the global accuracy of the model. The key to the success of adaptive sampling is therefore the selection of infill criteria, which determines the location of the new samples in the sample space. Wang et al.^37^ presented a novel adaptive sampling method based on the hyper-volume iteration (HVI) strategy for constructing surrogate aerodynamic models. Applying an adaptive sampling strategy based on expected local errors, Xu et al.^38^ proposed an ensemble of adaptive surrogate models. Rosenbaum et al.^39^ compared different sampling strategies and new theoretical methods using a dense set of validation data to gain a deeper understanding of optimal sample distributions and lower error boundaries. Based on Proper Orthogonal Decomposition (POD), Guenot et al.^40^ proposed a novel contribution to adaptive sampling strategies for non-intrusive reduced order models. In addition, adaptive sampling also applies infill criteria such as minimizing the predicted objective function (MP), maximizing the expected improvement function (EI)^41^, maximizing the probability of improvement function (PoI)^2^, minimizing the lower confidence bound (LCB)^42^, maximizing the mean squared error (MSE)^2^, and so on.

Currently, most adaptive sampling methods are designed for single-output problems. However, the surrogate model of a dam is usually a multi-outputs model with multiple related outputs. In general, the multi-outputs problem is comprised of asymmetric multi-outputs problem^43–45^ and symmetric multi-outputs problem^46^. As for the asymmetric multi-outputs problem, the simulation models with different computational costs and fidelities describe the same output. In contrast, for a symmetric multi-outputs problem, when simulating a set of inputs, different outputs are calculated simultaneously. In establishing the dam surrogate model, the structural behavior at different locations is anisotropic. The structural behavior of the dam is affected by the spatial location, but the input parameters are fixed. Therefore, the establishment of a dam surrogate model can be considered as a symmetric multi-outputs problem.

This article starting with the adaptive sampling method based on the RBFM. It applies to creating a surrogate model with multi-outputs using three infill criteria and entropy weight method. It then proposes an adaptive sampling method with multi-outputs based on RBFM. The method starts with a small initial sample set and establishes surrogate models for multi-outputs, respectively. Then, three infill criteria are used to search for infill samples, with MSE and EI being used for global and local searches, respectively. The ideal infill group is determined using the entropy weight method. Under the combination of these three infill criteria and entropy weight method, the adaptive sampling model can not only further sampling, make RBFM more accurate, but also solve the problem of multi-outputs sampling and improve the computational efficiency even further.

The remaining sections of this paper are structured as follows. In section “The proposed approach”, the proposed method's procedure is described in detail. In section “Benchmark function test”, benchmark functions demonstrate the applicability of the proposed method to SIMO and MIMO problems. Using typical measuring points, Section "Numerical experiment" establishes a surrogate model for numerical simulation of dams. Section "Conclusion" draws some conclusions.

## The proposed approach

This section introduces the surrogate model with multi-outputs based on the adaptive sampling strategy in detail, including the Design of Experiments (DoE), surrogate model, infill criteria, the determination of the ideal infill group with multi-outputs, stopping criteria, and the flow chart of the proposed method.

### Design of Experiments (DoE)

DoE^47^ is used to generate samples in the design space to establish the initial surrogate model. In order to obtain an accurate surrogate model with limited initial samples, uniform sampling is usually adopted for sample space. Sobol' quasi-random sequences, also known as “low-discrepancy” sequences, have the property of uniform distribution in space. Unlike random numbers, quasi-random points are deterministic sequences that know the position of previously sampled points and are constructed to avoid the presence of clusters as much as possible. Meanwhile, Sobol' quasi-random sequences stratify the following three main requirements: (1) Best uniformity of distribution as $N \to \infty$. (2) Good distribution for fairly small initial sets. (3) A very fast computational algorithm. Therefore, this paper takes Sobol sequence as initial sampling.

### Surrogate model

RBFM is a kind of nonlinear scatter interpolation model that fits the high dimensional function with the expression of one dimension function.

Suppose *n* samples are randomly taken as the training set, denoted as $\left( {x_{i} ,y_{i} } \right)$. Then the RBFM can be expressed as a linear combination, as shown in Eq. (1).

1 $${y\left( x \right)} = \sum\limits_{i = 1}^{n} {{\omega_{i}} \varphi \left( {\left\| {x - x_{i} } \right\|} \right)}$$

where $\varphi$ is the corresponding kernel function, $r_{i} = \left\| {x - x_{i} } \right\|$ represents the Euclidean distance between the sampling point $x$ of any parameter and the known sampling point $x_{i}$. $\omega_{i}$ is the weight coefficient of the kernel function at different training points. Common radial basis kernel functions include the Thin-plate spline function, Gaussian function, Multiquadric (MQ) function, Inverse multiquadric (IMQ), and so on. In this paper, Multiquadric (MQ) function is taken as the kernel function, and its expression is shown in Eq. (2).

2 $$\varphi \left( r \right) = \sqrt {r^{2} + c^{2} }$$

where *c* is the shape parameter of the radial basis function, whose value determines the specific shape of the kernel function.

Equation (1) of the above *n* sampling points can be used to obtain the system of equations shown in Eq. (3), from which the value of the weight coefficient $\omega_{i}$ can be obtained.

3 $$\left\{ \begin{gathered} \omega_{1} \varphi \left( {\left\| {x_{1} - x_{1} } \right\|} \right) + \omega_{2} \varphi \left( {\left\| {x_{1} - x_{2} } \right\|} \right) + \cdots + \omega_{n} \varphi \left( {\left\| {x_{1} - x_{n} } \right\|} \right) = y_{1} \hfill \\ \omega_{1} \varphi \left( {\left\| {x_{2} - x_{1} } \right\|} \right) + \omega_{2} \varphi \left( {\left\| {x_{2} - x_{2} } \right\|} \right) + \cdots + \omega_{n} \varphi \left( {\left\| {x_{2} - x_{n} } \right\|} \right) = y_{2} \hfill \\ \, \vdots \hfill \\ \omega_{1} \varphi \left( {\left\| {x_{n} - x_{1} } \right\|} \right) + \omega_{2} \varphi \left( {\left\| {x_{n} - x_{2} } \right\|} \right) + \cdots + \omega_{n} \varphi \left( {\left\| {x_{n} - x_{n} } \right\|} \right) = y_{n} \hfill \\ \end{gathered} \right.$$

Defining the coefficient vector ${\text{w}} = \left[ {\omega_{1} ,\omega_{2} , \ldots ,\omega_{n} } \right]^{T}$ and the matrix ${\Phi }_{i,j} = \varphi \left( {\left\| {x_{i} - x_{j} } \right\|} \right)$, where $i = 1,2, \ldots ,n$ and $j = 1,2, \ldots ,n$, this can be written as ${\Phi w} = y^{T}$. Then, provided the inverse of ${\Phi }$ exists, the matrix ${\text{w}} = {\Phi }^{ - 1} y^{T}$ can represent the above expression . The predictor of RBFM can be expressed as

4 $$\widehat{y}_{{N + 1}} = {\varvec{\upvarphi}} {\mathbf{w}} = {\varvec{\upvarphi}} {\varvec{\Phi}} ^{{ - 1}} y^{T}$$

where $$\varphi = \left[ {\varphi \left( {\left\| {x_{N + 1} - x_{1} } \right\|} \right),\varphi \left( {\left\| {x_{N + 1} - x_{2} } \right\|} \right), \ldots ,\varphi \left( {\left\| {x_{N + 1} - x_{N} } \right\|} \right)} \right]$$.

The RBFM treats that each deterministic response is the realization of some stochastic process (taken here to be a Gaussian random variable). Using the (Gaussian) distributions of *N* responses $y = \left[ {y_{1} ,y_{2} , \ldots ,y_{N} } \right]$ collected so far, it can be shown that the mean and the variance of the assumed stochastic process at $x_{N + 1}$ are^48^

5 $$\widehat{y}_{{N + 1}} = {\varvec{\upvarphi}} {\varvec{\Phi}} ^{{ - 1}} y^{T}$$

6 $$\sigma _{{\widehat{y}_{{N + 1}} }}^{2} = 1 - {\varvec{\upvarphi}} {\varvec{\Phi}} ^{{ - 1}} {\varvec{\upvarphi}} ^{T}$$

The variance of the Gaussian distribution (Eq. 6) will be taken as a measure of the likely error at the prediction points.

### Infill criteria

The infill criteria are developed to evaluate the prediction uncertainty and improvement of a current best value in global optimization by treating an unknown output as a realization of a stochastic process. In the proposed method, three infill criteria are used for self-adaptive sampling: (1) Mean squared error (MSE), (2) Expected improvement on minimum (EI_min_), (3) Expected improvement on maximum (EI_max_). Accordingly, three new points can be obtained at each updating cycle. The new sampling data is added to the initial DoE to update the surrogate models, which drives DoE towards the global optimum. The infill group above is based on a single-output. How to choose the ideal infill group in the case of multi-outputs is explained in section “Determine the ideal infill group with multi-outputs”.

#### Maximizing the expected improvement (EI)

Excepted improvement (EI) is an infill criterion to evaluate how much improvement of the current RBFM is expected if a new sample is obtained. We assume a random variable $Y\sim N\left[ {\hat{y}\left( x \right),s^{2} \left( x \right)} \right]$, where $\hat{y}$ is the RBFM predictor defined in Eq. (5), and *s*^2^ is the MSE defined in Eq. (6). Denoting the best objective value from the sample evaluated so far by $y_{\min } = \min \left[ {y_{1} ,y_{2} , \ldots ,y_{N} } \right]$, where, *y*_*min*_ is the minimum value of all current samples. Then the improvement on the minimum *I* can be defined as $I = y_{\min } - Y\left( x \right)$, where, *Y*(*x*) is the Gaussian distribution. The goal is to find a sample on *Y*(*x*) that makes $I > 0$. Equation (7) can be integrated to compute the expectation of *I*. So the expected improvement is given by

7 $$E\left[ {I\left( x \right)} \right]_{\min } = \left\{ {\begin{array}{*{20}c} {\left( {y_{\min } - \hat{y}} \right)\Psi \left( {\frac{{y_{\min } - \hat{y}}}{s}} \right) + s\psi \left( {\frac{{y_{\min } - \hat{y}}}{s}} \right)} & {if\;s > 0} \\ {0\quad \quad \quad \quad \quad \quad \quad \quad \quad \quad \quad \quad \quad \quad \;\;\,} & {if\;s = 0} \\ \end{array} } \right.$$

where $\Psi \left( \cdot \right)$ and $\psi \left( \cdot \right)$ are the cumulative distribution and probability density function of a standard normal distribution, respectively.

Equation (7) shows the EI on minimum, while EI on maximum is also applied to the selection of the infill points. Expected improvement on maximum can be expressed as

8 $$E\left[ {I\left( x \right)} \right]_{\max } = \left\{ {\begin{array}{*{20}c} {\left( {\hat{y} - y_{\max } } \right)\Psi \left( {\frac{{\hat{y} - y_{\max } }}{s}} \right) + s\psi \left( {\frac{{\hat{y} - y_{\max } }}{s}} \right)} & {if\;s > 0} \\ {0\quad \quad \quad \quad \quad \quad \quad \quad \quad \quad \quad \quad \quad \quad \quad \,} & {if\;s = 0} \\ \end{array} } \right.$$

Schonlau^49^ points out the expected improvement will tend to be large at a point with a predicted value smaller than *y*_min_ and/or there is much uncertainty associated with the prediction. Therefore, expected improvement can be considered as a balance between seeking promising areas of the design space and the uncertainty in the model.

#### Maximizing the mean squared error (MSE)

It can be found that in a high uncertainty region, the sparsity of training samples will lead to greater uncertainty of prediction of unknown samples. As a measure of the sparseness of the input space, MSE can be used as an infill criteria to search the infill sample that has a large prediction uncertainty. The MSE criteria is the prediction variance (*s*^2^) of the RBFM, so the infilling point $\left( {x_{MSE}^{n + 1} } \right)$ can be chosen by maximizing the MSE as

9 $$x_{MSE}^{n + 1} = \mathop {\arg \max s^{2} \left( x \right)}\limits_{x}$$

### Determine the ideal infill group with multi-outputs

According to the three infill criteria in section “Infill criteria”, adding a new infill group to the DoE can somewhat improve the quality of the surrogate model. However, when faced with a multi-outputs problem, we will get *m* infill groups (assuming there are *m* outputs). How to determine the ideal infill group among these *m* infill groups is the focus of the section. As an objective weighting method, entropy weight method can calculate the weight of each output and provide a reference for determining the ideal infill group. The detailed steps are as follows:

*Step 1* Establish a standard decision matrix. For the problem of *m* alternatives (outputs) and *n* attribute values (infill criterion), the initial decision matrix is:

10 $$A = \left( \begin{gathered} y_{11} \cdots y_{1n} \hfill \\ \, \vdots \, \ddots \, \vdots \hfill \\ y_{m1} \cdots y_{mn} \hfill \\ \end{gathered} \right)$$

The initial matrix *A* is normalized:

11 $$B = \left( \begin{gathered} b_{11} \cdots b_{1n} \hfill \\ \, \vdots \, \ddots \, \vdots \hfill \\ b_{m1} \cdots b_{mn} \hfill \\ \end{gathered} \right)$$

where *b*_ij_ is the standard values of the *j*th evaluation indicator in *i*th alternatives.

The formula for normalization is:

12 $$b_{ij} = \frac{{y_{ij} }}{{\sum\limits_{i = 1}^{m} {y_{ij} } }}$$

In the proposed method, the columns represent MSE, EI_min_ and EI_max_, respectively.

*Step 2* Calculate the weights based on the entropy weight method.

Define the entropy of *j*th indicators

13 $$e_{j} = - k\sum\limits_{i = 1}^{n} {p_{ij} \ln p_{ij} }$$

where

14 $$p_{ij} = \frac{{b_{ij} }}{{\sum\limits_{i = 1}^{n} {b_{ij} } }},k = \frac{1}{\ln \left( n \right)}$$

Since the smaller the entropy, the larger is the weight, the weight for the *j*th indicators is:

15 $$w_{j} = \frac{{1 - e_{j} }}{{\sum\limits_{j = 1}^{n} {\left( {1 - e_{j} } \right)} }}$$

where $$w_{j} \in \left[ {0,1} \right]$$, $$\sum\limits_{j = 1}^{n} {w_{j} = 1}$$, $$j = 1,2, \ldots ,n$$.

*Step 3* Calculate the close degree *T*_i_.

The weighting matrix *Z* is defined as:

16 $$Z = \left( \begin{gathered} z_{11} \cdots z_{1n} \hfill \\ \, \vdots \, \ddots \, \vdots \hfill \\ z_{m1} \cdots z_{mn} \hfill \\ \end{gathered} \right) = \left( \begin{gathered} w_{1} b_{11} \cdots w_{n} b_{1n} \hfill \\ \, \vdots \, \ddots \, \vdots \hfill \\ w_{1} b_{m1} \cdots w_{n} b_{mn} \hfill \\ \end{gathered} \right)$$

Determine the ideal solution matrix *P*:

17 $$P = \left[ {p_{1} ,p_{2} , \ldots ,p_{n} } \right]$$

where $$p_{j} = \mathop {\max z_{ij} }\limits_{j}$$, $$j = 1,2, \ldots ,n$$.

Calculate the close degree *T*_i_:

18 $$T_{i} = 1 - \frac{{\sum\limits_{j = 1}^{n} {z_{ij} p_{j} } }}{{\sum\limits_{j = 1}^{n} {\left( {p_{j} } \right)^{2} } }}$$

It can be analyzed that the bigger the value of *T*, the better is the solution. The ideal infill group can be determined by the maximum value of *T*, and the determined infill group will be added to the DoE.

### Stopping criteria

In order to evaluate the performance of surrogate model and determine the stopping criteria of adaptive sampling, it is necessary to select an appropriate index.

R-squared value (R^2^) and root mean square error (RMSE) are the most commonly used evaluation indexes, which are often used to evaluate the performance of surrogate model. The formula is as follows:

19 $$R^{2} = 1 - \frac{SSE}{{SST}} = 1 - \frac{{\sum\limits_{i = 1}^{n} {\left( {y_{i} - \hat{y}_{i} } \right)^{2} } }}{{\sum\limits_{i = 1}^{n} {\left( {y_{i} - \overline{y}} \right)^{2} } }}$$

20 $$RMSE = \sqrt {\frac{{\sum\limits_{i = 1}^{n} {\left( {y_{i} - \hat{y}_{i} } \right)^{2} } }}{n}}$$

where SSE is the sum of squares of residuals, SST is the total sum of squares. $\hat{y}_{i}$ and *y*_i_ denote the prediction value from the surrogate model and true output at the inferred samples. $\overline{y}$ is the average value of *y*.

R^2^ is commonly used to measure how well a surrogate model explains changes in the data. R^2^ has a great ability to explain the linear correlation of the model, but tends to be weak when facing complex nonlinear problems. As the standard deviation of the residual between the response value and the predicted value, RMSE commonly pays more attention to the degree of the surrogate model’s prediction of the absolute value of the response value. This paper focuses more on the prediction results of the surrogate model. However, when the prediction error is too large, it will have a great influence on RMSE, especially facing variables with larger dimensions. Therefore, the normalized root mean square error (NRMSE) is used as the model accuracy evaluation index to verify the prediction of the surrogate model. NRMSE is defined as follows:

21 $$NRMSE = \sqrt {\frac{1}{n}\sum\limits_{i = 1}^{n} {\left( {\frac{{y_{i} - \hat{y}_{i} }}{\max \left( y \right) - \min \left( y \right)}} \right)^{2} } }$$

For multi-outputs problems, each output corresponds to an NRMSE. According to “short board effect theory”^50^, the overall accuracy of a multi-outputs problem is determined by the worst model accuracy, so the definition of the system accuracy index NRMSE is:

22 $$NRMSE_{sys} = \mathop {\max }\limits_{1 \le j \le m} \left\{ {NRMSE_{j} } \right\}$$

### Steps for the proposed approach

**Figure 1:**
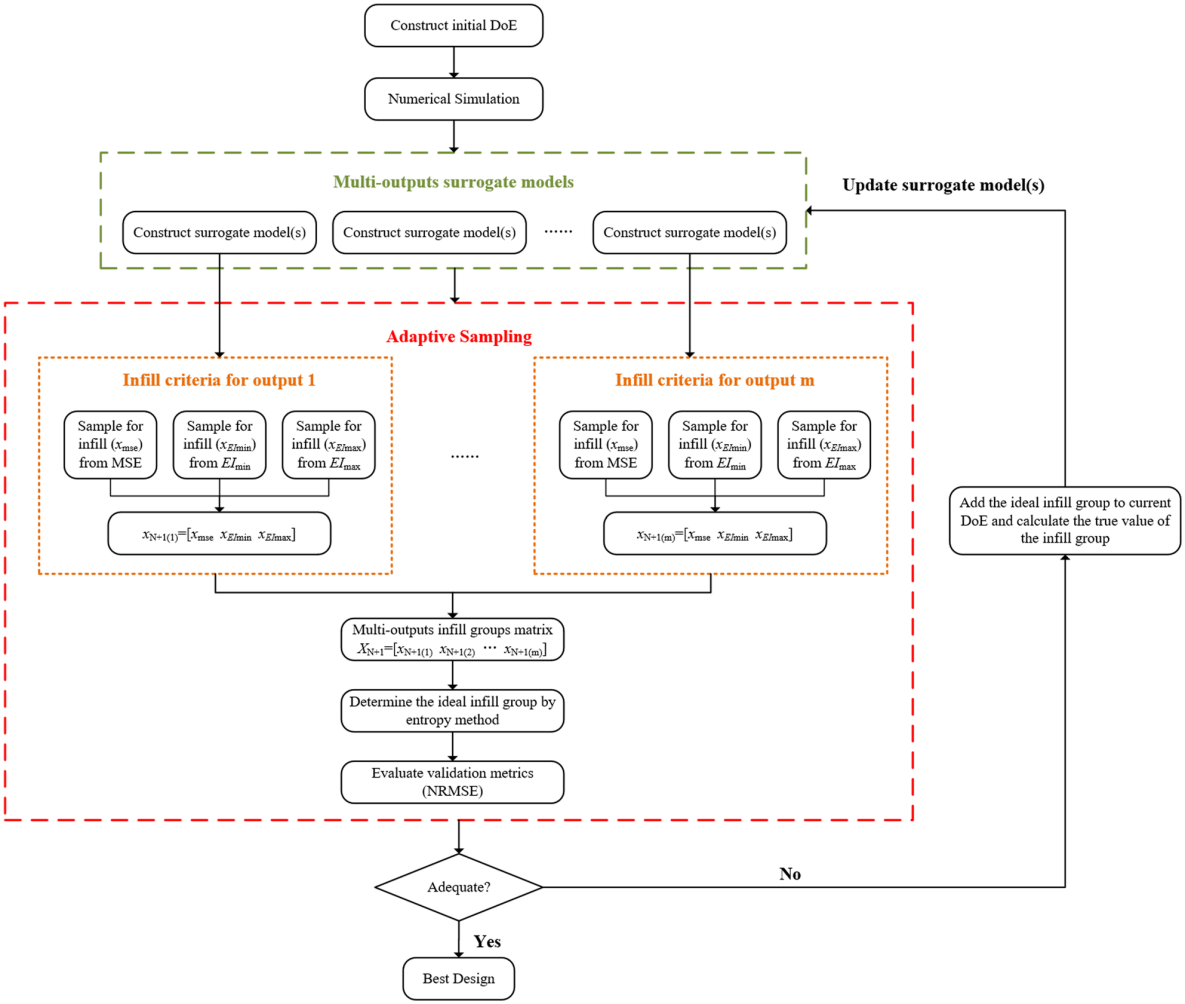
The flowchart of the proposed approach.

The flow chart of the proposed method is shown in Fig. 1, and the specific implementation steps are as follows:

1. Generate the initial DoE with Sobol sequence.
2. Calculate the real date by numerical simulation.
3. Establish surrogate models with multi-outputs.
4. Three infill criteria are used to select infill groups for different outputs to form a multi-outputs infill groups matrix.
5. Calculate the close degree of each infill group to determine the ideal infill group.
6. Calculate the validation metrics (NRMSE) based on validation set.
7. Estimate whether the validation metrics satisfy the stopping criteria. If satisfied, the calculation will be stopped. Otherwise, the ideal infill group will be added to DoE and repeat step (3) until satisfy the stopping criteria.

## Benchmark function test

This section uses two kinds of benchmark functions to verify the applicability and stability of the adaptive sampling method with multi-outputs in single-input & multi-outputs (SIMO) and multi-inputs & multi-outputs (MIMO) problems. Meanwhile, the modeling accuracy of the surrogate model with multi-outputs is analyzed by NRMSE.

### Single-input & multi-outputs (SIMO) problem

In this section, the proposed adaptive sampling method with multi-outputs is applied to SIMO problem, and the applicability of the method is investigated. Here, a non-stationary benchmark function proposed by Gramacy et al.^51^ and a multi-model benchmark function are used to verify this. For benchmark function *f*_1_, due to the addition of periodic terms, the benchmark function has a certain fluctuation. Meanwhile, when x increases to a certain extent (*x* ≥ 2), the exponential term will increase greatly, which will gradually reduce the fluctuation of the function. For benchmark function *f*_2_, the fluctuation degree of the benchmark function gradually increases with the increase of *x* due to the addition of a linear function. For these two benchmark functions, benchmark function *f*_1_ has a strong fluctuation when *x* is small, while *f*_2_ has a strong fluctuation when *x* is large. Therefore, the proposed adaptive sampling method can be well tested using these two benchmark functions. The specific formula is as follows:

23 $$f_{1} = \frac{{\sin \left( {10\pi x} \right)}}{2x} + \left( {x - 1} \right)^{4} ,x \in \left[ {0.5,2.5} \right]$$

24 $$f_{2} = - \left( {1.4 - 3x} \right)\sin \left( {18x} \right),x \in \left[ {0.5,2.5} \right]$$

**Figure 2:**
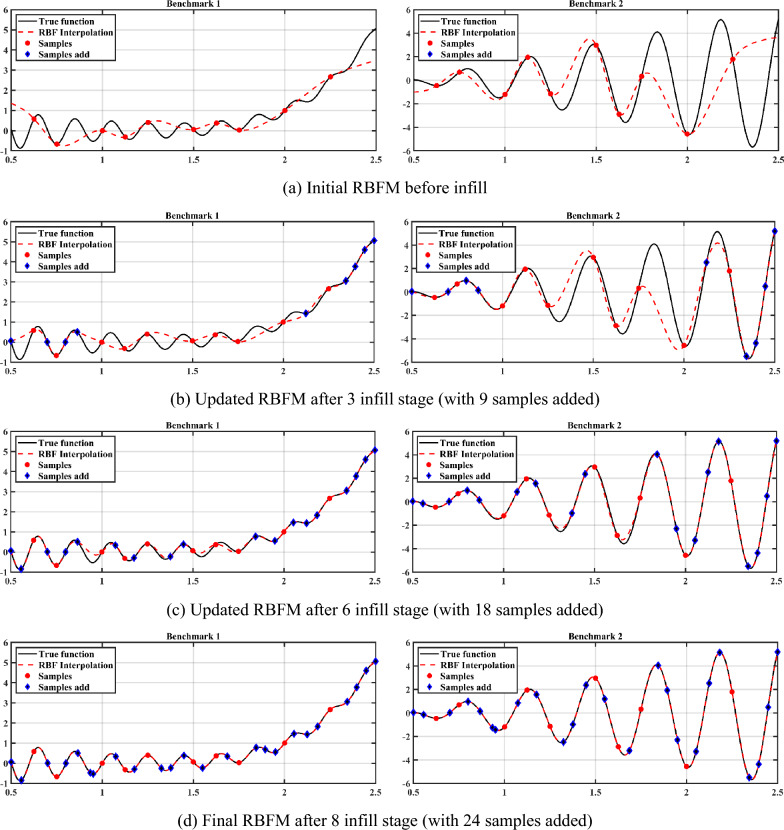
The application of adaptive sampling with multi-outputs to SIMO problem.

For DoE, 10 random samples are generated by Sobol sequence. It is very difficult to choose the stopping criteria value, and a suitable stopping criteria can prevent overfitting or underfitting. This paper refers to the selection of stopping criteria in different literatures^52,53^, and finally determines that the stopping criteria is 0.01. This section also gives the calculation process figure and NRMSE after each adaptive sampling, as shown in Fig. 2 and Table 1, respectively. The ideal infill group for each stage is shown in bold.

**Table 1:**
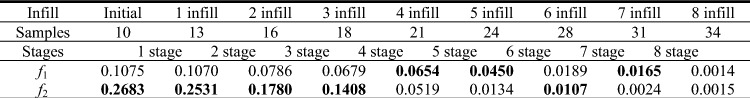
NRMSE after each stage of adaptive sampling.

**Figure 3:**
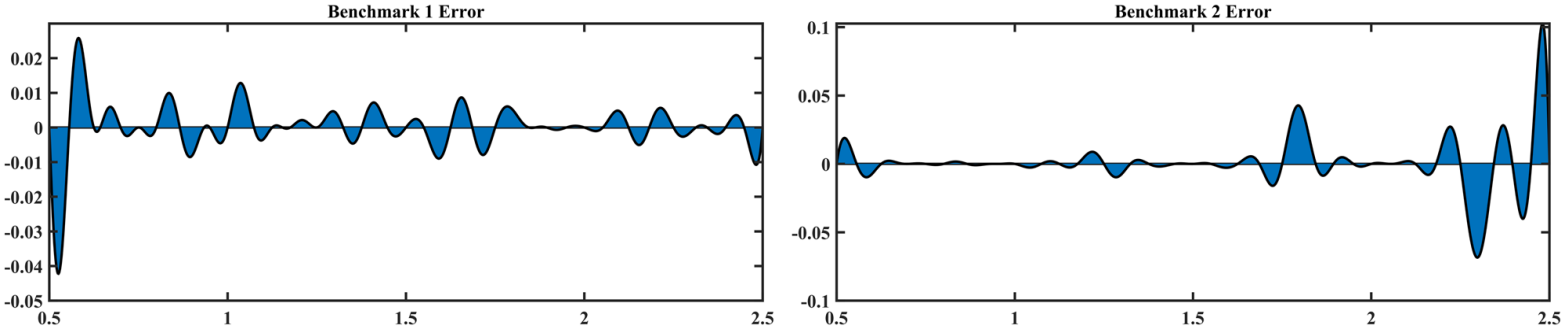
Model error corresponding to final surrogate model.

For the benchmark function in SIMO problem, the proposed adaptive sampling method runs 8 stages, and eventually sampled a total of 34 samples, including 10 initial samples and 24 infill samples. Combined with the analysis of Fig. 2 and Table 1, the following conclusions can be drawn:

1. Under the initial sample size, the NRMSE of *f*_1_ and *f*_2_ are 0.1075 and 0.2683, respectively. As can be seen from the figure, due to the insufficient initial sample size and the adoption of random sampling method, the initial RBFM cannot reflect the maximum and minimum values of the original model exactly, which makes the performance of the surrogate model unsatisfactory.
2. After 3 stages of adaptive sampling, the current DoE consists of 10 initial samples and 9 infill samples. Compared with the initial model, the performance of the model is greatly improved, and the NRMSE of *f*_1_ and *f*_2_ decrease from 0.1075 and 0.2683 to 0.0679 and 0.1408. It can be found that the model accuracy improvement of *f*_2_ is significantly greater than that of *f*_1_, because the infill group corresponding to *f*_2_ is selected as the ideal infill group in the first 3 stages. The newly added 9 infill samples are mainly clustered at *x* ≤ 1 and *x* ≥ 2, which significantly improves the model performance of surrogate model of *f*_2_. While for *f*_1_, although the model performance has been improved to a certain extent, there are still some differences with the original model (Supplementary Information).
3. After 6 stages of adaptive sampling, a total of 18 infill samples are added. It can be found that *f*_1_ and *f*_2_ are basically similar to the original model under this stage, and NRMSE is decreased to 0.0189 and 0.0107, respectively. In stage 4, infill samples corresponding to *f*_2_ are selected for the ideal infill group, while infill samples corresponding to *f*_1_ are selected for stage 5 and 6. By observing the newly added 9 infill samples, it can be found that most of them are at the maximum and minimum values of the benchmark functions, which makes the surrogate model more closed to original model.
4. After 8 stages of adaptive sampling, NRMSE of the two benchmark functions satisfy the stopping criteria, which is 0.0014 and 0.0015, respectively. At this point, the figure of the surrogate model is basically the same as the original model. Combined with the model error figure in Fig. 3, it can be found that the model error of *f*_2_ is slightly larger than that of *f*_1_, with the maximum error reaching 0.1, while that of *f*_1_ is only 0.04. This is because normalized MSE is used to measure the model performance. Assuming another stage of adaptive sampling, the model error of *f*_2_ will be further reduced. In stage 7, the infill samples corresponding to *f*_2_ are selected as the ideal infill group. At this point, NRMSE of *f*_2_ has satisfies the stopping criteria, so *f*_2_ is no longer selected as the ideal infill group in stage 8.
5. According to Table 2, it can be found that when Sobol sequence samples 34 samples, NRMSE of *f*_1_ and *f*_2_ are 0.0015 and 0.0023, larger than NRMSE corresponding to adaptive sampling. When the size of samples reaches 40, the surrogate model performance of *f*_1_ is better than that of the proposed method, while the surrogate model performance of *f*_2_ is not significantly improved. This is because in Sobol sequence, samples generated by quasi-random sequence are not targeted at large errors, so the samples are considered as a waste of computational performance and invalid sampling. Until the sample size reaches 47, Sobol sequence obtain valid sample. Therefore, the adaptive sampling method with multi-outputs proposed in this paper can save 27.66% of sampling cost.
6. In conclusion, the adaptive sampling method with multi-outputs plays a well performance in SIMO problem. This method can carry out targeted samples according to the characteristics of the function and can greatly reduce the sample size required to train the surrogate model.

**Table 2:** Comparison of NRMSE of adaptive sampling with multi-outputs and Sobol sequence in SIMO problem.

Samples	RBFM			Sobol sequence				
	34	34	……	40	……	45	46	47
*f*_1_	0.0014	0.0015	……	0.0010	……	0.0009	0.0009	0.0008
*f*_2_	0.0015	0.0023	……	0.0020	……	0.0020	0.0020	0.0001

### Multi-inputs & multi-outputs (MIMO) problem

**Figure 4:**
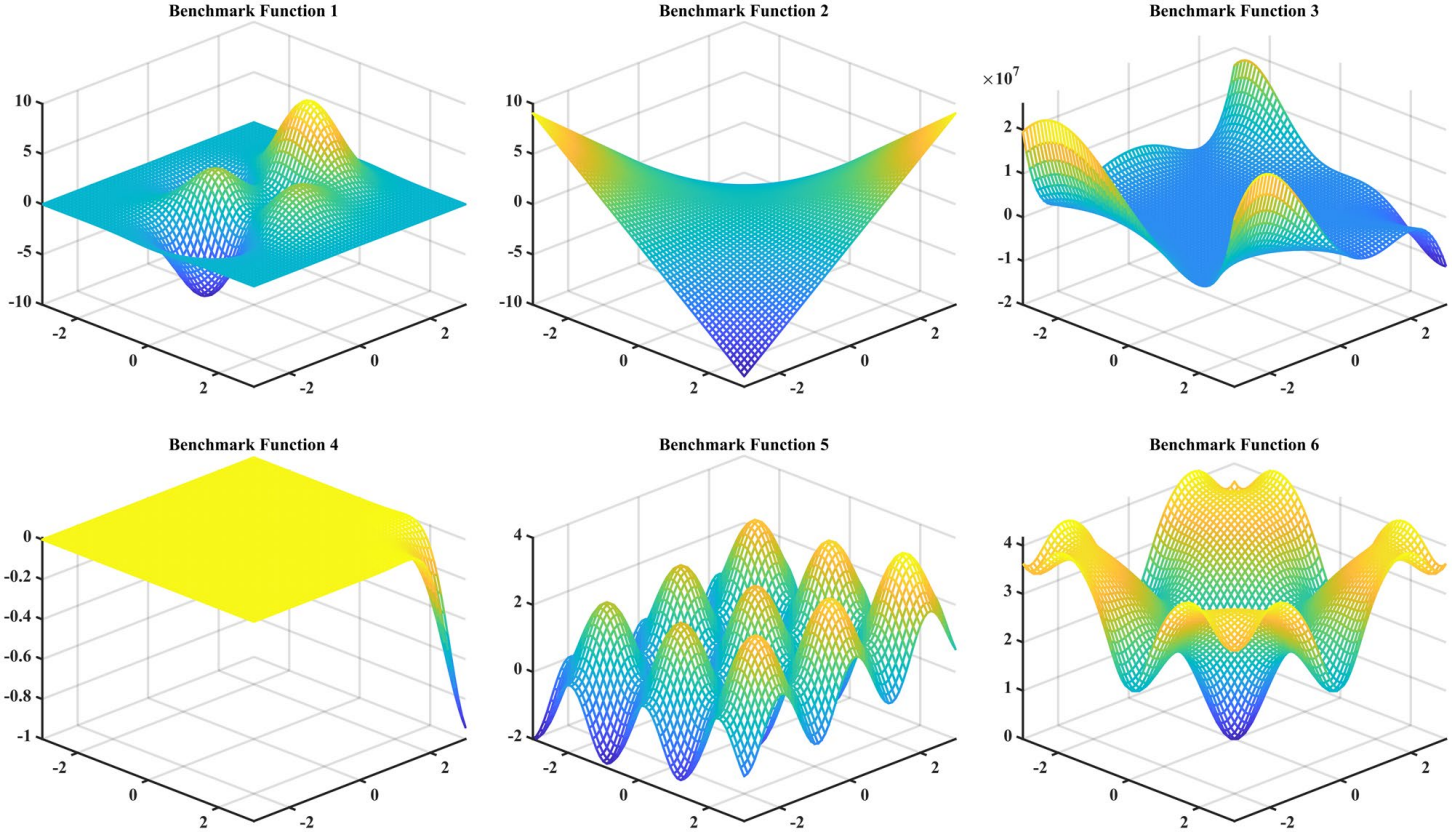
Figure of benchmark functions in MIMO problem.

The previous section analyzed the application of the adaptive sampling method with multi-outputs in SIMO problem. This section will focus on the application of this method in MIMO problem. In MIMO problem, after the surrogate model is established for each output and its infill group is obtained, the ideal infill group is determined based on the entropy weight method to update the DoE. On this basis, the surrogate model for each output is reconstructed. This process is repeated until the surrogate model for each output meets the stopping criteria. As the sample size increases, the accuracy of the surrogate model for each output tends to increase. However, the ideal infill group can improve the accuracy of the surrogate model corresponding to the output. This section verifies the method using six benchmark functions in Liu et al.^46^. The figures of these six benchmark functions are shown in Fig. 4, and the specific formula is as follows:

Benchmark Function 1 (BF1)

25 $$\begin{aligned} f_{1} = & 3\left( {1 - x_{1} } \right)^{2} \exp \left( { - x_{1}^{2} - \left( {x_{2} + 1} \right)^{2} } \right) \\ \, & - 10\left( {\frac{{x_{1} }}{5} - x_{1}^{3} - x_{2}^{5} } \right)\exp \left( { - x_{1}^{2} - x_{2}^{2} } \right) \\ \, & - \frac{1}{3}\exp \left( { - \left( {x_{1} + 1} \right)^{2} - x_{2}^{2} } \right),x_{1,2} \in \left[ { - 3,3} \right] \\ \end{aligned}$$

Benchmark Function 2 (BF2)

26 $$f_{2} = x_{1} x_{2} + 0.05,x_{1,2} \in \left[ { - 3,3} \right]$$

Benchmark Function 3 (BF3)

27 $$\begin{aligned} f_{3} = & \left( {1 + \left( {x_{1} + x_{2} + 1} \right)^{2} \left( {19 - 14x_{1} + 13x_{1}^{2} - 14x_{2} + 6x_{1} x_{2} + 3x_{2}^{2} } \right)} \right) \\ \, & \left( {30 + \left( {2x_{1} - 3x_{2} } \right)^{2} \left( {18 - 32x_{1} + 12x_{1}^{2} - 48x_{2} - 36x_{1} x_{2} + 27x_{2}^{2} } \right)} \right), \\ \, & x_{1,2} \in \left[ { - 3,3} \right] \\ \end{aligned}$$

Benchmark Function 4 (BF4)

28 $$\begin{aligned} f_{4} = & - \cos \left( {x_{1} } \right)\cos \left( {x_{2} } \right)\exp \left( { - \left( {x_{1} - \pi } \right)^{2} - \left( {x_{2} - \pi } \right)^{2} } \right), \\ \, & x_{1,2} \in \left[ { - 3,3} \right] \\ \end{aligned}$$

Benchmark Function 5 (BF5)

29 $$\begin{aligned} f_{5} = & \frac{1}{36}\left( {\left( {x_{1} + 3} \right)^{2} + \left( {x_{2} + 3} \right)^{2} } \right) \\ \, & - \left( {\cos \left( {3\left( {x_{1} + 3} \right) + \cos \left( {3\left( {x_{2} + 3} \right)} \right)} \right)} \right), \\ \, & x_{1,2} \in \left[ { - 3,3} \right] \\ \end{aligned}$$

Benchmark Function 6 (BF6)

30 $$\begin{aligned} f_{6} = & - 20\exp \left( { - \frac{{x_{1}^{2} + x_{2}^{2} }}{90}} \right) \\ & - \exp \left( {0.5\left( {\cos \left( {\frac{2\pi }{3}x_{1} } \right) + \cos \left( {\frac{2\pi }{3}x_{2} } \right)} \right)} \right) + 20 + \exp \left( 1 \right), \\ & x_{1,2} \in \left[ { - 3,3} \right] \\ \end{aligned}$$

As can be seen from Fig. 4, these six functions have different characteristics. The benchmark function 1 (BF1) contains multiple Gaussian and polynomial terms with different centers. Thus, it has a multi-modal region in the center and a flat boundary region. The benchmark function 2 (BF2) has a simple second-order polynomial term. Compared to BF2, the benchmark function 3 (BF3) has a higher-order polynomial term, and performs a highly nonlinear responses near the boundaries. Besides, its output value is much larger than the output values of other functions. The benchmark function 4 (BF4) has a Gaussian function centered at $\left( {\pi ,\pi } \right)$ and some periodic terms. Therefore, except for a single peak in the right corner, it produces almost zero output across the entire region. The output of the benchmark function 5 (BF5) waving uniformly within the region. The benchmark function 6 (BF6) has a Gaussian function located in the center and another Gaussian function with some periodic terms. Therefore, it produces a single peak region and a boundary region where the output value changes uniformly at the center.

Similar to Sect.  3.1, for DoE, 50 random samples are generated by Sobol sequence, and for stopping criteria, 0.01 is set here. For the benchmark function in MIMO problem, the proposed adaptive sampling method runs 16 stages, and eventually sampled a total of 98 samples, including 50 initial samples and 48 infill samples. The table below shows the NRMSE calculated for each stage and the ideal infill group selected for each stage, where the ideal infill group for each stage is shown in bold. Table 4 compares the results of the adaptive sampling method with multi-outputs and Sobol sequence. The following conclusions can be drawn by combing Tables 3 and 4:

1. Under the initial sample size, the NRMSE of each benchmark function are 0.026429, 0.000026, 0.009237, 0.013803, 0.062813, and 0.041767. Only *f*_2_ and *f*_3_ satisfy the stopping criteria. Compared with the other four benchmark functions, *f*_2_ and *f*_3_ have simpler forms and therefore require smaller sample sizes. While for the other four benchmark functions, the initial DoE is insufficient to cover all characteristics, so adaptive sampling is required.
2. During the 16 stages of adaptive sampling, the ideal infill groups mainly focused on *f*_1_, *f*_5_, and *f*_6_. After each infill, it can be found that the NRMSE of the benchmark functions corresponding to the ideal infill group is basically better than other benchmark functions. When the adaptive sampling reaches stage 8, the NRMSE of *f*_1_ satisfies the stopping criteria. Therefore, *f*_1_ is no longer used as an indicator of high close degree in the subsequent selection of ideal infill group. Similarly, in stage 13, NRMSE of *f*_6_ also satisfies the stopping criteria. At this time, only *f*_5_ does not satisfy the stopping criteria. Therefore, the last 4 stages of adaptive sampling are targeted on *f*_5_.
3. For the six benchmark functions in MIMO problem, this section also establishes a surrogate model using Sobol sequence and compares the results with the adaptive sampling with multi-outputs. The proposed method satisfies the stopping criteria at 98 samples, while Sobol sequence satisfies the stopping criteria at 150 samples. Compared with Sobol sequence, this method can save more than 30% of sample cost. The NRMSE of these two methods on 98 samples are 0.006339, 0, 0.000277, 0.000769, 0.008146, 0.009856 and 0.026285, 0.000022, 0.007969, 0.013974, 0.059359, 0.040626. It can be found that the NRMSE of Sobol sequence is significantly higher than that of the proposed method. Similar to SIMO problem, random sampling fails to sample the model in a targeted way, leading to the waste of a large number of samples.
4. In conclusion, the adaptive sampling method with multi-outputs plays a well performance in MIMO problem. Moreover, sampling can be adjusted according to the function form, which greatly reduce the cost of model calculation.

**Table 3:**
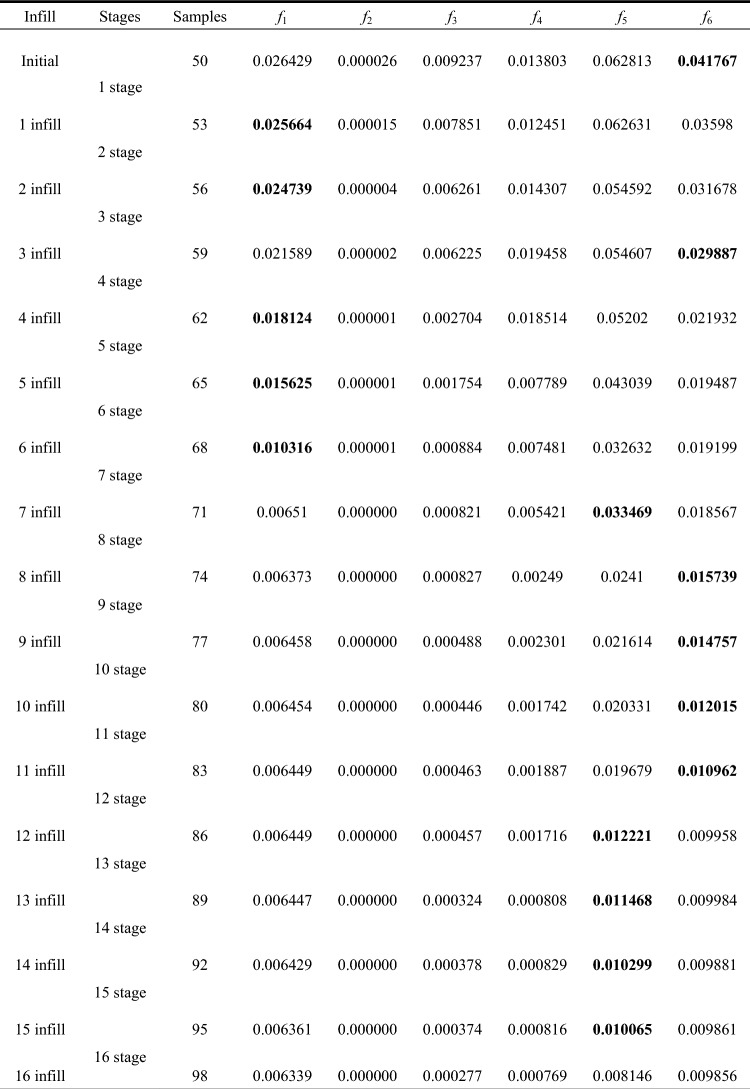
Calculation process of adaptive sampling with multi-outputs in MIMO problem.

**Table 4:** Comparison of NRMSE of adaptive sampling with multi-outputs and Sobol sequence in MIMO problem.

Samples	RBFM	Sobol sequence								
	98	98	99	100	……	125	……	148	149	150
*f*_1_	0.006339	0.026285	0.026193	0.024832	……	0.014458	……	0.003218	0.003127	0.003115
*f*_2_	0.000000	0.000022	0.000021	0.000021	……	0.000002	……	0.000000	0.000001	0.000001
*f*_3_	0.000277	0.007969	0.007648	0.007478	……	0.001524	……	0.000719	0.000718	0.000565
*f*_4_	0.000769	0.013974	0.014340	0.014194	……	0.007028	……	0.006778	0.007791	0.006958
*f*_5_	0.008146	0.059359	0.058255	0.057947	……	0.023823	……	0.006914	0.007422	0.004905
*f*_6_	0.009856	0.040626	0.040539	0.040628	……	0.021781	……	0.011062	0.010068	0.009582

## Numerical experiment

In this section, adaptive sampling with multi-outputs is applied to a gravity dam. The dam is located on the Brahmaputra River in Tibet, China. The controlled catchment area of dam toe is 157,407 km^2^, the annual average flow is 1010 m^3^/s, the total reservoir capacity is 57.89 million m^3^, the normal water level of the reservoir is 3477.00 m, and the corresponding reservoir capacity is about 55.28 million m^3^. The dam is an RCC gravity dam with a maximum dam height of 118.0 m and a total crest length of 389 m. It is divided into 17 sections, among which 6^#^–9^#^ is the overflow dam section and the rest is the water retaining dam section.

**Figure 5:**
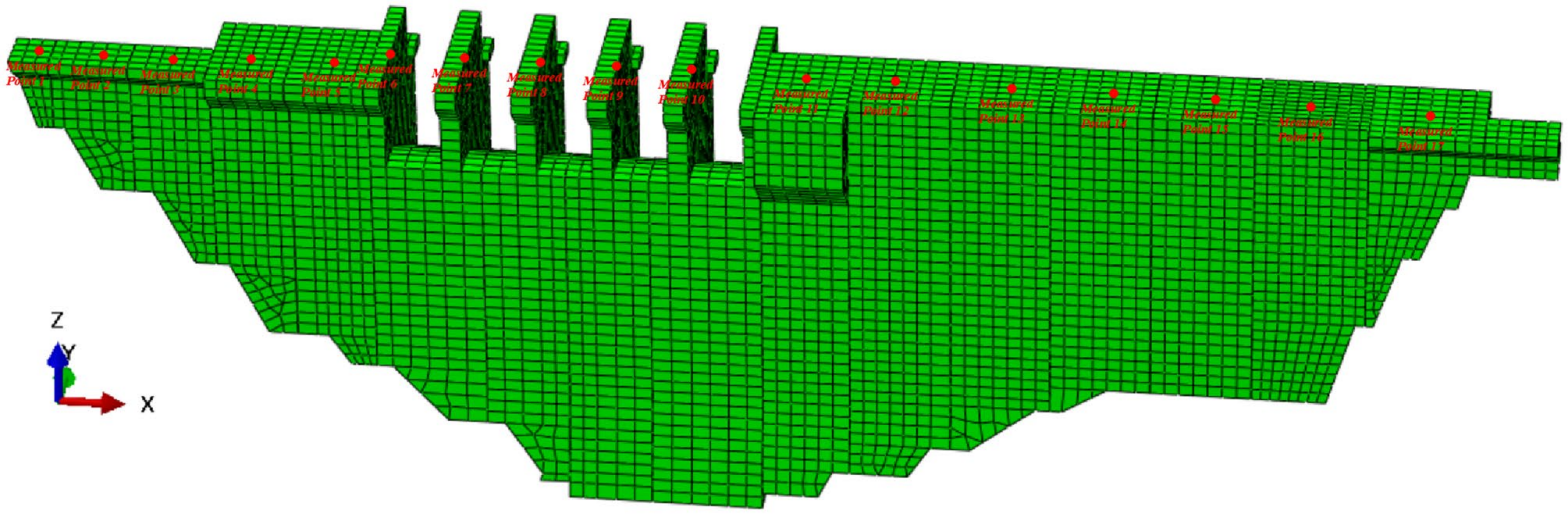
Figure of layout of measuring points.

In this paper, 17 typical measuring points are selected from the top of the dam, and the dam surrogate model of these 17 measuring points are established by using the adaptive sampling with multi-outputs. The layout of measuring points is shown in Fig. 5.

For DoE, 100 random samples are generated by Sobol sequence in this section. Variable information is shown in Table 5. In engineering problems, the nonlinearity of the model is more complex than that of the benchmark function. When the NRMSE of the model increases to 0.05, every improvement will be slow and will pay a lot of calculation cost. Combined with previous engineering experience, NRMSE is set to 0.05 in most cases. And for engineering problems, setting NMRSE to 0.05 can relatively meet the requirements of most cases. Therefore, NRMSE is set to 0.05 here. Aiming at this practical engineering problem, the proposed adaptive sampling method runs 7 stages, and eventually sampled a total of 121 samples, including 100 initial samples and 21 infill samples. Similar to section “Benchmark function test”, the NRMSE calculated for each stage and the ideal infill group selected for each stage are also given here, where bold represents the ideal infill group for each stage, as shown in Table 6.

**Table 5:** Variable information table for adaptive sampling of gravity dam.

ID	Parameter	Symbol	Unit	Value range
1	Time	*t*	*month*	[0,12]
2	Upstream water level	$H_{1}$	m	[3340,3451]
3	Downstream water level	$H_{2}$	m	[3350,3390]
4	Uplift reduction coefficient of upstream curtain	$\alpha_{1}$	–	[0.1,0.8]
5	Uplift reduction coefficient of downstream curtain	$\alpha_{2}$	–	[0.1,0.8]
6	Elastic modulus of concrete	$E$	GPa	[10, 30]
7	Linear expansion coefficient of concrete	$\alpha_{C}$	–	[1e−6,1e−5]
8	Thermal conductivity of concrete	$\lambda_{C}$	${\text{kJ}}/\left( {{\text{mh}}^\circ {\text{C}}} \right)$	[3, 10]
9	Deformation modulus of rock	$E_{0}$	GPa	[5, 30]

**Table 6:**
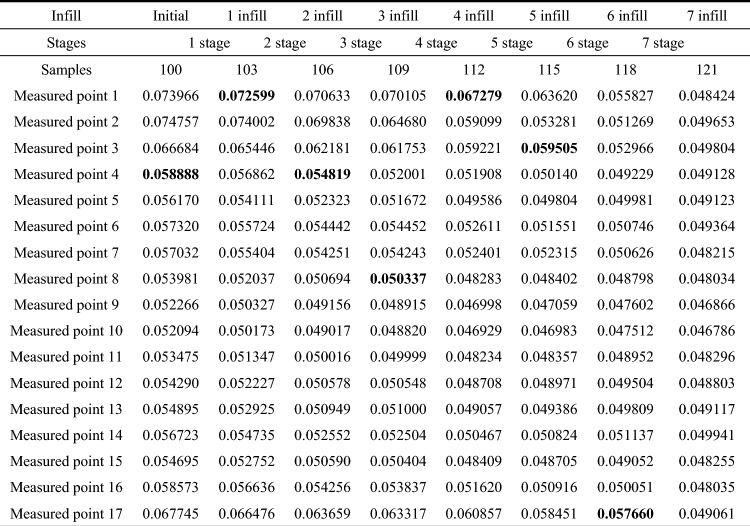
Calculation process of adaptive sampling with multi-outputs in practical engineering problem.

It can be found that under the initial DoE, NRMSE of each benchmark function is less than 0.1. However, in some benchmark functions, NRMSE is close to 0.05. Therefore, whether the selection of 100 initial samples is too much for the surrogate model of dam based on typical measuring points will be discussed later. In the process of 7 stages of adaptive sampling, although the ideal infill group may not be located at the measuring point with the maximum error of each stage, but it will always select the measuring point with large error. After each stage of infill, the NRMSE of the measuring point corresponding to the ideal infill group tends to decrease significantly.

In order to verify that this method requires fewer samples than uniform sampling or random sampling for multi-outputs problems, this paper uses random sampling to generate 1000 samples and performs the simulations. 200 samples are selected as the verification set, while the other 800 samples are compared with adaptive sampling method. The results are shown in Table 7.

**Table 7:** Comparison of NRMSE of adaptive sampling with multi-outputs and Sobol sequence in practical engineering problem.

Samples	RBFM	Sobol sequence								
	121	100	……	121	……	398	399	400	……	800
Measured point 1	0.048424	0.081990	……	0.075488	……	0.047336	0.046714	0.046687	……	0.040520
Measured point 2	0.049653	0.084527	……	0.079405	……	0.050023	0.049959	0.049844	……	0.038861
Measured point 3	0.049804	0.076409	……	0.070937	……	0.044292	0.044088	0.044010	……	0.035276
Measured point 4	0.049128	0.067418	……	0.060653	……	0.037925	0.037397	0.037374	……	0.031483
Measured point 5	0.049123	0.064906	……	0.057471	……	0.035937	0.03531	0.035296	……	0.030091
Measured point 6	0.049364	0.070815	……	0.061677	……	0.038747	0.037695	0.037695	……	0.033120
Measured point 7	0.048215	0.070380	……	0.060946	……	0.038635	0.037638	0.037638	……	0.032926
Measured point 8	0.048034	0.063730	……	0.055298	……	0.034942	0.034199	0.034195	……	0.029425
Measured point 9	0.046866	0.062352	……	0.053715	……	0.034215	0.033503	0.033502	……	0.028701
Measured point 10	0.046786	0.062254	……	0.053616	……	0.034113	0.033387	0.033386	……	0.028637
Measured point 11	0.048296	0.063534	……	0.055283	……	0.035573	0.034838	0.034832	……	0.029893
Measured point 12	0.048803	0.064131	……	0.056028	……	0.035944	0.035204	0.035192	……	0.030442
Measured point 13	0.049117	0.064550	……	0.056687	……	0.036163	0.035427	0.035406	……	0.030547
Measured point 14	0.049941	0.066314	……	0.058485	……	0.037100	0.036351	0.036325	……	0.031322
Measured point 15	0.048255	0.063542	……	0.055886	……	0.035417	0.034801	0.034771	……	0.029698
Measured point 16	0.048035	0.067454	……	0.060208	……	0.037666	0.037176	0.037141	……	0.031041
Measured point 17	0.049061	0.078435	……	0.072831	……	0.046113	0.045401	0.045360	……	0.038574

It can be seen from Table 7 that when the stopping criteria is set to 0.05, 121 samples are selected by the adaptive sampling with multi-outputs. While when Sobol sequence randomly sampled 121 samples, every measuring point is greater than stopping criteria, but compared with 100 samples, the performance of the model is improved. The performance of the surrogate model does not satisfy the stopping criteria until the sample size of Sobol sequence reaches 400. It can be found that in the previous sampling process, the rest of the measuring points all satisfied the stopping criteria, however, due to the characteristics of random sampling, the surrogate model of measuring point 2 does not been well improved. When the sample size of Sobol sequence reaches 800, it can be found that although the performance of the surrogate model has been improved to some extent, but considering the high calculation cost, this part of sampling can be considered redundant and meaningless.

In the analysis of Table 6, we mentioned that 100 initial samples are too much to establish a surrogate model of dam based on typical measuring points. Therefore, in order to explore the influence of the initial sample size on the final sample size, adaptive sampling is used for different initial sample size. The result is shown in Table 8.

**Table 8:** The influence of different initial sample size on the final sample size.

	Sampling number							
Initial sampling	2	10	20	30	……	80	90	100
Finial sampling	95	97	101	105	……	107	108	121

As can be seen from Table 8, only 95 samples are required when 2 initial samples are selected, while 121 samples are required when 100 initial samples are selected. With the increase of the initial sample size, the final sample size required by this method also increased gradually. Whether different initial sample size will affect the distribution form of the final sampling results of each variable will be studied in detail below.

**Figure 6:**
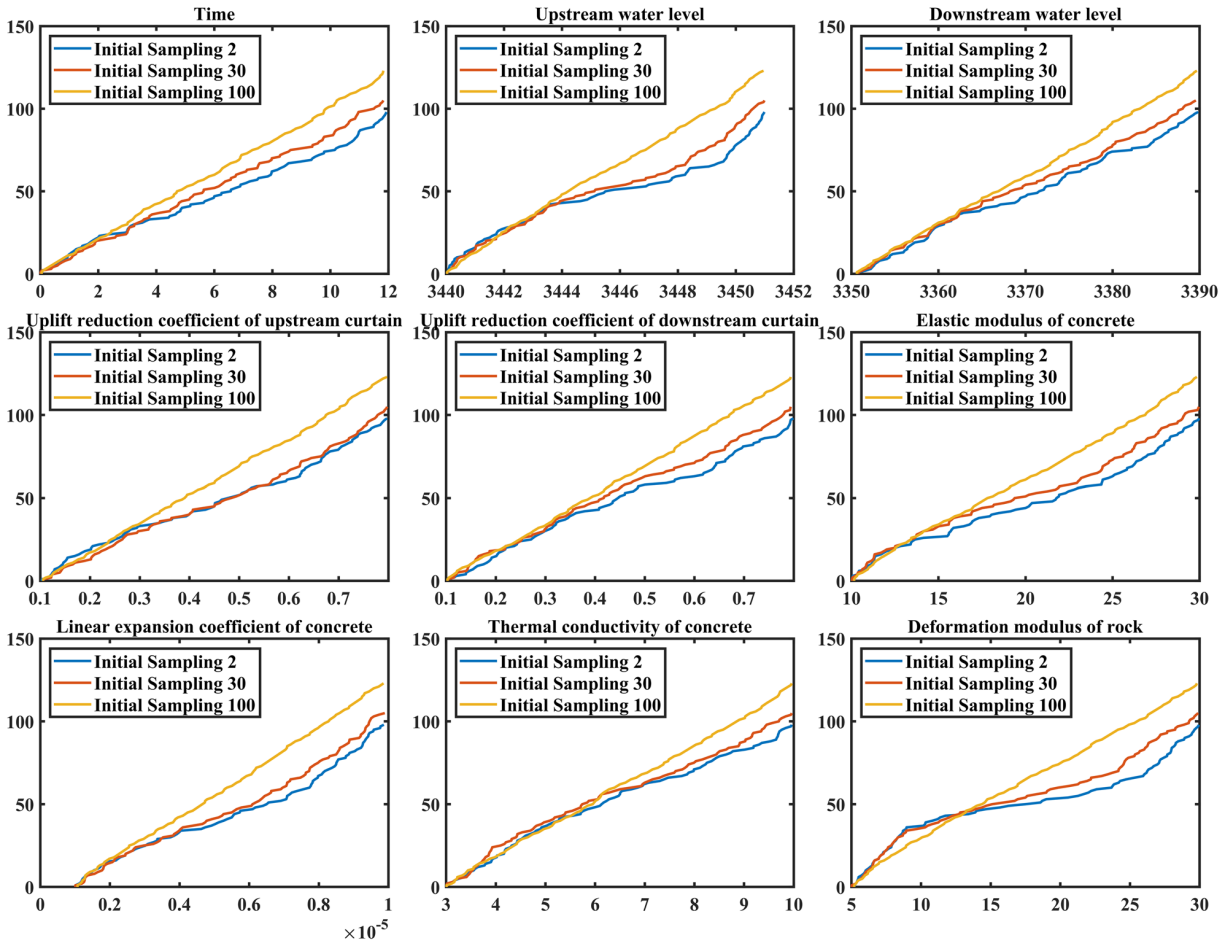
The influence of different initial sample size on distribution form of final sampling results of each variable.

Figure 6 shows the influence of different initial sample sizes on the distribution form of the final sampling results of each variable. 2, 30, and 100 initial samples are selected as examples. As can be seen from the figure, when the initial sample is 100, all variables tend to be uniformly distributed on the whole, but there are still some slight fluctuations in some areas. However, when the initial samples are 2 and 30, the sample size of some variables will increase within a certain value range. And the rules of these two initial samples are the same.

For the time in variable space, the distribution form of this variable after the final sampling is basically not correlated with the initial sample size, and the distribution form presents a uniform distribution state. This rule is reasonable because the uniformity of time is important to the model. For the upstream water level in variable space, it can be obviously found that the sample size increases significantly when the water level is high. Elastic modulus and linear expansion coefficient of concrete perform similar rules. For the thermal conductivity of concrete in variable space, more samples are gathered at low thermal conductivity. For the deformation modulus of rock in variable space, when it is in the range of 10–25, the samples are few. The remaining variables are not significantly influenced by the initial sample size. Based on the above analysis, it can be found that different initial sample sizes have a certain impact on the final sample sizes, and the final sample size will increase with the increase of the initial sample size, which also reflects from the side that the proposed method can carry out targeted samples according to different forms of surrogate models.

## Conclusion

Based on the adaptive sampling technique, the application of the adaptive sampling technique in establishing the surrogate model with multi-outputs is discussed. Then, SIMO and MIMO problems are studied based on the benchmark functions and compared with Sobol sequence. The result shows that this method has a smaller sample size requirement. Finally, by establishing a surrogate model of dam numerical simulation with multi-outputs, the applicability of this method in practical engineering problems is explored. In addition, the influence of different initial sample sizes on the final sample size and the distribution form of final sampling results of each variable are also analyzed. The following conclusions are obtained:

1. Since the adaptive sampling model with multi-outputs is developed based on the Bayesian framework, samples can be targeted according to the function form of surrogate model. In addition, by comparing with Sobol sequence, it is found that this method can effectively reduce the sample size required to establish the surrogate model.
2. Different initial sample size has a certain influence on the final sample size. Take this practical engineering problem as an example, the smaller the initial sample size, the smaller the final sample size. The smaller the initial sample size, the earlier the adaptive sampling process, and the more targeted samples can be carried out. Therefore, when the initial sample size is small, a clustering phenomenon will occur in some variables within a certain value range.
3. In order to verify the applicability of the proposed method, this method is applied to SIMO and MIMO problems, respectively. The result shows that this method can adaptively select the weights of different outputs and determine the ideal infill group, which greatly reduces human intervention. In addition, compared with random sampling, it can be found that this method can use fewer samples to establish a more accurate surrogate model.
4. In addition to SIMO and MIMO problems, this method is also applied to establish the surrogate model of dam numerical simulation with multi-outputs. The adaptive sampling based on multiple measuring points can be effectively applied to the parameter back analysis of dam structureto improve the computational efficiency of the back analysis.
5. It should be noted that the proposed method is currently applied to the problem in 17 dimensions, and problems with higher dimensions have not been verified. When facing higher dimension problems, the curse of dimensionality is still challenging. Therefore, the focus of future research will be how to conduct adaptive sampling with multi-outputs for higher dimension problems.

## Supplementary Information

Supplementary Information.
